# Characterization of the Different Chemical Components and Nutritional Properties of Two *Eryngium* Species

**DOI:** 10.3390/foods14010118

**Published:** 2025-01-03

**Authors:** Mozhgan Roudbari, Mohsen Barzegar, Esther Sendra, Isabel Casanova-Martínez, Marcos Rodríguez-Estrada, Ángel A. Carbonell-Barrachina

**Affiliations:** 1 Department of Food Science and Technology, Faculty of Agriculture, Tarbiat Modares University, Tehran P.O. Box 14155-336, Iran; roudbari.m@modares.ac.ir; 2 Research Group of Food Quality and Safety, Instituto de Investigación e Innovación Agroalimentaria y Agroambiental (CIAGRO-UMH), Universidad Miguel Hernández de Elche, Ctra. Beniel, km 3.2, 03312 Orihuela, Spain; esther.sendra@umh.es (E.S.); isabel.casanova@goumh.umh.es (I.C.-M.); marcos.rodrigueze@umh.es (M.R.-E.); angel.carbonell@umh.es (Á.A.C.-B.)

**Keywords:** *Eryngium billardieri*, *Eryngium planum*, polyphenols, fatty acids, minerals, antioxidant, herbal tea

## Abstract

This study aimed to investigate the nutritional value and potential for herbal tea production of two species *Eryngium*. The analysis includes the quantification of lipids, proteins, organic acids (HPLC-MS), sugars (HPLC-MS), phenolic compounds (HPLC-MS-MS), volatile compounds (GC-MS), fatty acids (GC-MS), amino acids (HPLC-MS-MS), some minerals (ICP-MS), total phenolic content, and antioxidant activities of *Eryngium billardieri* flowers (EBF) and thorns (EBT), as well as *Eryngium planum* flowers (EPF) and thorns (EPT). The results indicate that EPF and EPT exhibit elevated levels of protein (11.2%) and sugars (224.2 mg/gdw), respectively. Whereas, EBF demonstrates a higher concentration of amino acids (7.13 mg/100 gdw) and total phenolic content (19.25 mg GAE/gdw), which correlates with pronounced antioxidant properties. Oleic acid was notable in *E. billardieri*, while linoleic and α-linolenic acids were predominant in *E. planum*. Furthermore, essential minerals such as Fe, Mn, Zn, Mg, K, Ca, and P were also determined. Sensory evaluations by panelists confirmed that tea derived from the studied species possesses favorable taste and flavor profiles, attributed to its rich volatile compounds. These findings highlight the nutritional value of *Eryngium* species as a functional ingredient in the food industry. Additionally, their antioxidant properties suggest promising uses in pharmaceutical applications.

## 1. Introduction

In recent years, there has been an increasing focus on the use of natural resources, like plants, which are often available and have minimal side effects for potential applications in medicine and the food industry. People are increasingly interested in adopting healthier lifestyles, including consuming foods that offer health benefits beyond basic nutrition. Natural sources rich in phytochemicals exhibit antimicrobial, antioxidant, and anti-inflammatory properties [1]. More than 30,000 edible plant species found in natural habitats have mostly been ignored and not given much attention for their potential use in food and medicine [2].

The genus *Eryngium*, belonging to the apiaceae family, is recognized as the largest and most complex genus within this family, with approximately 250 species distributed across various regions of the world [3]. *Eryngium* species are valued for their diverse applications and ornamental, culinary, agricultural, and medicinal uses.

In medicinal uses, *E. billardieri*, an herb native to Iran, is widely used in traditional medicine for its therapeutic properties. It is effective in treating inflammatory conditions such as rheumatism and sinusitis, as well as for wound healing and urinary infections [4]. Additionally, it is used for treating arthritis pain, relieving constipation, and managing diabetes [5]. Also, E. *planum*, in European and Asian traditional medicine, is extensively used for its medicinal properties [6]. It is used for its calming effects in treating hemorrhoids, rheumatic diseases, inflammation, and heartburn [7].

The phytochemical components of these plants include polyacetylenes, flavonoids, saponins, coumarins, and monoterpene glycosides [8].

Recently, studies have been conducted on the bioactive properties of *E. billardieri* and *E. planum*. For example, Daneshzadeh et al. [4] demonstrated that the ethanolic extract of *E. billardieri* contains a total phenolic content ranging from 10.71 to 33.38 mg gallic acid equivalent/gdw of extract, along with total flavonoids between 15.04 and 27.13 mg quercetin equivalent/gdw of extract. The antioxidant activity of this extract varied from 17.25% to 51.63%. Additionally, the extract showed inhibitory and/or control effects on bacteria and fungi. In another study, the essential oil of *E. billardieri*, primarily containing n-hexadecanoic acid, as well as 2-pentadecanone, 6, 10, 14-trimethyl, 1H-indene, 1-ethylideneoctahydro-, and cinnamyl tiglate, has been shown to effectively inhibit 24 tested gram-negative and gram-positive bacteria. The minimum inhibitory concentrations range from 0.67 to 34.17 g L^−1^ [5]. In a related study, the extract obtained by ultrasound-assisted extraction from *E. planum* showed a diversity of polyphenolic compounds, mainly flavonoids (rutin and isoquercitrin) [6]. Additionally, the extraction of essential oil from *E. planum* has been conducted using various methods, such as hydrodistillation, ultrasound-assisted hydrodistillation, and headspace solid-phase microextraction (HS-SPME). Their results showed that β-copaene, a sesquiterpene hydrocarbon, was the predominant compound in the *E. planum* oil obtained by both hydrodistillation and ultrasound-assisted hydrodistillation methods. Cis-chrysanthenyl acetate, an oxygenated monoterpene, was identified as the major compound within the *E. planum* volatiles extracted by the HS-SPME. Additionally, these essential oils had notable antimicrobial activity against pathogens like *E. coli* and *S. aureus* [9]. Moreover, Mahmoudi et al. [10] investigated the effect of different drying methods on the essential oil content and composition of *E. planum*. Chromatographic analysis showed that the main components of the essential oil of this plant included β-elemene, α-pinene, trans-β-farnesene, cis-chrysanthenyl acetate, and germacrene A. Various drying techniques significantly influenced these main components as well as the total phenolic and flavonoid content. The highest phenolic content (66.62 mg GAE/gdw of extract) was observed under specific infrared drying conditions, while the highest total flavonoid content (6.5 mg quercetin equivalent/gdw of extract) and the greatest antioxidant capacity (IC_50_ 192.66 µg/mL) were associated with oven drying.

The existing literature predominantly focuses on the extraction methodologies for the essential oils of *E. billardieri* and *E. planum*, with particular emphasis on their phytochemical compositions and potential antimicrobial properties. However, a review of the existing literature reveals a notable scarcity of studies focused on nutritional compounds, particularly chemical compositions including lipid, protein, ash, fiber, volatile compounds, amino acids, fatty acids, sugars, organic acids, macro- and microelements, and the phenolic content of these plants extract. Furthermore, most of these studies have focused on the aerial parts, leaves, and roots of the *Eryngium* genus, while the thorn has been never investigated.

The identified gaps in the literature present a significant opportunity for further investigation into the nutritional compounds of these species. This research aims to enhance our understanding of the potential contributions of the flower and thorn constituents of these two species, focusing on their applications as functional ingredients and potential flavor enhancers in food products. Additionally, this study lays the groundwork for future work in the areas of nutrition, as well as the development of functional foods and pharmaceuticals derived from the two *Eryngium* species.

## 2. Materials and Methods

### 2.1. Plant Material

The taxonomic identification of two studied plants was conducted by the Research Institutes of Forests and Rangelands of Iran (Tehran, Iran). The aerial parts of the *E. planum* plant were harvested during the full flowering stage from a research farm located at Tarbiat Modares University (35°44′ N and 51°09′ E) in Tehran, Iran. The *E. billardieri* plant was harvested from the wild around Ganjnameh, Hamadan (34.7608° N, 48.4384° E), Iran. Samples were dried in a well-ventilated room in the shade and then stored in paper bags at 25 ± 2 °C.

### 2.2. Methods

#### 2.2.1. Proximate Chemical Composition

Chemical composition including moisture, ash, fat, and protein (Kjeldahl method using a conversion factor of 6.25) levels were analyzed according to the Association of Official Analytical Chemists methods [11]. Neutral detergent fiber (NDF), acid detergent fiber (ADF), and crude fiber contents were determined by using an ANKOM200/220 fiber analyzer (ANKOM Technology, Macedon, NY, USA) [12]. The available carbohydrates were calculated using Equation (1), as follows:

Carbohydrates (%) = 100 − (moisture + ash + fat + protein) %(1)

#### 2.2.2. Organic Acid and Sugar Analysis

Organic acids and sugars were identified and quantified, as described by Lipan et al. [13]. Each sample (0.5 g) was mixed with 10 mL of phosphate buffer 50 mM (pH = 7.8) and homogenized at 15,000× *g* for 1 min (Ultra-Turrax, T-25 Digital homogenizer). Then, samples were sonicated (Model 3000512, JP Selecta SA, Barcelona, Spain) with a constant frequency of 40 kHz at 20 °C for 15 min and centrifuged at 10,000× *g* for 10 min at 4 °C (Sigma 3–18 K; Osterode and Harz, Germany). The supernatant was filtered through a 0.45 μm Millipore filter before injection into a Hewlett-Packard HPLC series 1100 (Hewlett-Packard, Wilmington, DE, USA). This HPLC is equipped with a refractive index detector for sugar detection and a UV/Vis detector for organic acids analysis. A column (Supelcogel™ C-610H column 30 cm × 7.8 mm) and a pre-column (Supelguard 5 cm × 4.6 mm; Supelco, Bellefonte, PA, USA) were used for the analyses of both organic acids and sugars. The elution (run isocratically at 30 °C) buffer consisted of 0.1% phosphoric acid at a flow rate of 0.5 mL/min and organic acids’ absorbance was measured at 210 nm. These same HPLC conditions (elution buffer, flow rate, and column) are used for the analysis of sugars. The quantification of organic acids, as well as sugars, was conducted using an external standard method. The analysis was performed in triplicate and the findings are presented as mean ± standard deviation in g/kg dw.

#### 2.2.3. Analysis of Fatty Acids

Fatty acids were quantified according to the method proposed by Clemente-Villalba et al. [14]. About 0.06 g of extract was mixed with 100 μL of dichloromethane and 1 mL of 0.5 M sodium methoxide in methanol and then placed in a hot water bath at 90 °C for 10 min. Subsequently, the samples were rapidly cooled in an ice bath. Next, 1 mL of methanolic boron trifluoride (BF_3_) was added and the mixture was placed in a dark place for 30 min. Following this incubation period, 1 mL of ultrapure water and 1 mL of hexane were added, and the samples were shaken for 2 min using a vortex mixer (Vortex 1, IKA, Staufen, Germany). Afterward, centrifugation was performed at 1500× *g* at 5 °C for 10 min. The supernatant was carefully recovered and placed in an amber chromatography vial. For separating compounds, a Shimadzu GC-2030 gas chromatograph coupled with a flame ionization detector (FID) and an automatic injector AOC-20i (Shimadzu Scientific Instruments, Inc., Columbia, MD, USA) was utilized. Helium was the carrier gas at a flow rate of 24 mL min^−1^. The FID utilized hydrogen and air at flow rates of 32 mL min^−1^ and 200 mL min^−1^, respectively. A Supelco SP^®^-2380 capillary column (60 m × 0.25 mm × 0.20 μm) was employed in the GC system. The injector temperature was 240 °C. The split ratio was set to 1:20, and the total linear flow velocity was 28.4 cm s^−1^. The temperature program started at 100 °C, which was held for 1 min. This was followed by a temperature increase at a rate of 3 °C min^−1^ until it reached 220 °C. Subsequently, the temperature was increased at a rate of 5 °C min^−1^ until it reached 245 °C, where it was held for 1 min. The detector temperature was maintained at 260 °C. The results were expressed as the percentage of each fatty acid in the total fatty acids profile.

#### 2.2.4. Elemental Analysis by ICP-MS

The determination of mineral concentrations was conducted following the method described by Clemente-Villalba et al. [14]. Each sample (0.5 g) was transferred into a digestion tube and treated with 8 mL of concentrated ultratrace quality nitric acid (69% *w*/*v*). A pre-digestion was performed for an hour. Afterward, 2 mL of ultratrace quality H_2_O_2_ 30% *w*/*v* was added, tubes were sealed, and samples were digested using a Milestone Ethos 1 microwave digester. Digestion was conducted for 15 min at 800 W. Following digestion, the samples were transferred to 10 mL volumetric flasks and made up to volume with ultrapure deionized water (18 MΩ Milli-Q^®^ system; Millipore Corporation, Madrid, Spain). Before analyzing the elements, samples were diluted 100-fold using ultrapure deionized water. The total concentrations of macroelements (Ca, K, Mg, Na, and P) and microelements (As, B, Cd, Cu, Fe, Mn, Pb, Se, and Zn) in the samples were quantified using an Inductively Coupled Plasma Mass Spectrometer (ICPMS-2030, Shimadzu Scientific Instrument, Inc., Columbia, MD, USA).

#### 2.2.5. Determination of Volatile Compounds

Volatile compounds were extracted using headspace solid-phase microextraction (HS-SPME) with a 50/30 µm divinylbenzene/carboxen/polydimethylsiloxane (DVB/CAR/PDMS) fiber (Supelco, Bellefonte, PA, USA). Subsequently, the extracted compounds were separated and quantified using a GC (Shimadzu GC2030) coupled with a Shimadzu TQ8040 NX mass spectrometer (Shimadzu Scientific Instruments, Inc., Columbia, MD, USA) in accordance with the methodology described by Anderica et al. [9].

#### 2.2.6. Identification and Quantification of Phenolic Compounds

The phenolic compound profiles of the samples were determined by the Agilent 1100 HPLC-ESI-DAD-MS-MS system (the mass spectrometer equipped with an ion trap analyzer). For the chromatographic method, a Poroshell 120 EC-C18 column (3 mm × 100 mm, 2.7 μm, Agilent Technologies) was used at a temperature of 25 °C. Samples (50 mg) were extracted with 1 mL of methanol 80% *v*/*v*, homogenized with a vortex mixer, and sonicated (Model 3000512, JP Selecta SA, Barcelona, Spain) with a constant frequency of 40 kHz for 30 min at room temperature. The samples were then centrifuged at 20,000× *g* for 15 min at 4 °C. The supernatant was filtered through a 0.22 μm membrane filter, with a sample injection volume of 5 μL. The mobile phases were water: formic acid (99:1, *v*/*v*) as phase A and acetonitrile as phase B, with a flow rate of 0.5 mL min^−1^. The gradient elution program was as follows: 5% B (0 min), 18% B (7 min), 28% B (17 min), 50% B (22 min), and 90% B (27–28 min), returning to initial conditions (5% B) at 29 min and maintained until 33 min. The diode array detector (DAD) was set to scan wavelengths from 200 to 600 nm with a scanning frequency of 2.5 Hz, and chromatograms were recorded at 320 and 360 nm. Nitrogen was used as the drying gas with a flow rate of 11 L min^−1^ at a temperature of 350 °C and as the nebulizing gas at a pressure of 65 psi. The capillary voltage was set to 4 kV. Mass spectra (MS) and fragments (MS-MS) were recorded in negative mode, in the range of 100–1500 *m*/*z*.

#### 2.2.7. Free Amino Acids Analysis

To separate and determine the concentration of free amino acids, a UPLC–MS/MS (model 8050, Shimadzu, Kyoto, Japan) was used. Approximately 50 mg of each sample was mixed with 200 μL of a 20 mM solution of 2-morpholinoethanesulfonic acid and 200 μL of ethanol. The mixture was subsequently shaken, centrifuged at 1500× *g* for 15 min, and filtered through a nylon membrane with a pore size of 0.45 μm. The filtrate was then diluted 500-fold with ethanol. External standards, with concentrations ranging from 0.0005 to 2.25 ppm, were employed for the quantification of amino acid concentrations [15].

#### 2.2.8. Total Phenolic Content (TPC) and Antioxidant Activity

TPC and antioxidant activity (ABTS^o+^, DPPH^o^, and FRAP) were conducted as described by Cano-Lamadrid et al. [16]. In the extraction step, 0.2 g of the samples were mixed with a 5 mL extracting solvent (Methanol/water (80:20, *v*/*v*) + 1% HCl).

For TPC analysis, 0.1 mL of the sample was mixed with 0.2 mL of Folin–Ciocalteau’s reagent and 2 mL of ultrapure water. This mixture was left in the dark place for 3 min. Then, 1 mL of sodium carbonate (20%) was added and allowed to stand at room temperature in darkness for 1 h. After the reaction period, absorbance was recorded at 765 nm.

To determine DPPH free radical scavenging activity, 10 μL of the sample was mixed with 950 μL of DPPH^o^ (100 μM) and 40 μL of methanol. After a 10-min incubation, the absorption was measured at 515 nm.

For preparing a solution of ABTS radical cation, ABTS (7.0 mM, 10 mL) and potassium persulfate (2.45 mM, 5 mL) were prepared in ethanol 10% and left to incubate in darkness at 20 °C for 16 h. Subsequently, the solution was diluted 100-fold in distilled water until its absorbance reached 0.70 ± 0.02 at 734 nm. To determine the radical scavenging activity, 0.99 mL of the ABTS^o+^ solution was mixed with 10 μL of the sample, and the absorbance was measured after 6 min at 734 nm.

The FRAP reagent was prepared by mixing acetate buffer solution (1.55 g L^−1^ of sodium acetate + 0.9 mL of HCl), TPTZ (10 mM in HCl (40 mM)), and FeCl_3_ (20 mM in ultrapure water) in a ratio of 10:1:1 (*v*/*v*/*v*). For the analysis, 0.99 mL FRAP reagent was mixed with 10 mL of sample and kept for 10 min before measuring the absorbance at 593 nm.

All measurements were conducted using a UV–Vis spectrophotometer (Helios Gamma model, UVG 1002E; Helios, Cambridge, UK) in triplicate.

#### 2.2.9. Infusion Process

Sixty samples of tea were prepared using mineral water and tea bags containing *E. billardieri* and *E. planum* flower parts (1, 2, and 3 g). Each tea bag was steeped in 250 mL of boiling water for varying durations (1, 2, 3, 4, and 5 min). Subsequently, the prepared herbal teas were cooled in an ice bath for color assessment and sensory analysis.

##### Color Analysis

Color was assessed using a CIEL*a*b* system and a Minolta CR200 colorimeter with D65 illuminant (Minolta Camera Co., Osaka, Japan). For the measurement, 20 mL of each formulation was placed in a quartz glass box specifically designed for liquid samples. The results were expressed as mean ± standard deviation of triplicate determinations.

##### Sensory Evaluation

After color analysis, 12 samples of teas were selected and sensory analysis was carried out by six experienced trained panelists (from the Department of Agro-Food Technology (UMH). Samples were presented to the panelists in coded clear plastic cups containing each tea. After training, the list of sensory attributes was assessed by the panelists (detailed in Table S1) in the visual phase, olfactory phase, and gustatory phase. A quantitative descriptive analysis test was designed and the intensity of perception was scored for each attribute on a scale ranging from 0 (very low) to 9 (very high).

#### 2.2.10. Statistical Analyses

All analyses were performed in triplicate, and the results were reported as mean ± standard deviation. To analyze the mean values, one-way ANOVA followed by Tukey’s HSD post hoc test was used. A *p*-value of 0.05 was considered statistically significant.

## 3. Results and Discussion

### 3.1. Proximate Composition

The proximate compositions of the flower and thorn parts of *E. billardieri* and *E. planum* are presented in Table 1. No significant differences were observed in moisture content (6.7–8.1%) across all samples (*p* > 0.05). The ash content of EPF (9.96%) was slightly higher than that of EBF (9.01%). Notably, EBT exhibited the lowest ash content at 4.94% (*p* < 0.05).

Protein content was significantly higher (*p* < 0.05) in EPF (11.2%) and EPT (10.4%) compared to EBF (8.3%) and EBT (4.5%). Tac et al. [17] reported that the protein contents in the aerial parts of *E. maritimum* and *E. campestre* were 13.97 and 10.77 g/100 g, respectively. Furthermore, EBF, EPF, and EPT did not exhibit statistically significant differences in fat content (*p* < 0.05). However, the EBT had the lowest fat content (*p* < 0.05).

Table 1 also presents data on crude fiber, NDF, and ADF, which are important parameters for assessing total cell wall and lignocellulose content. The EBF exhibited slightly lower levels of crude fiber, NDF, and ADF compared to EPF. Notably, the EBT displayed the highest concentrations of crude fiber (50.9%), NDF (71.8%), and ADF (48.8%) among the analyzed samples. This increased content may be related to the higher presence of thorns in the *E. billardieri* variety. Thorny structures may comprise a significant portion of the cell wall, which plays a critical role in providing strength and rigidity to the plants. Previous research has shown that these components constitute a substantial fraction of a plant’s dry weight, primarily consisting of cellulose, hemicellulose, and lignin [18].

### 3.2. Organic Acid and Sugar Profiles

The concentrations of organic acids in the analyzed samples are shown in Table 2. The findings indicate that quinic acid and malic acid exhibited the highest concentrations, while tartaric acid displayed the lowest levels among the samples. There was no statistically significant difference in the concentrations of quinic acid between the EPF (16.0 mg/gdw) and EBF (16.4 mg/gdw) samples (*p* > 0.05); however, the EPT (13.4 mg/gdw) had a higher concentration of quinic acid compared to the EBT (12.2 mg/gdw). Furthermore, there were no significant differences in the levels of citric acid among the EPF (8.3 mg/gdw), EPT (8.6 mg/gdw), and EBF (7.2 mg/gdw) samples; nonetheless, the EBT sample (4.7 mg/gdw) exhibited the lowest citric acid concentration (*p* < 0.05). De la Luz Cádiz-Gurrea et al. [19] also reported the presence of quinic, malic, and citric acids in *E. bourgatii* extract.

In terms of sugar composition, the EBF sample (160.9 mg/gdw) demonstrated a lower sugar content than the EPF sample (196.3 mg/gdw). Fructose was identified as the predominant sugar, with concentrations of 72.5 (EPF), 79.6 (EPT), 56.5 (EBF), and 44.8 mg/gdw (EBT). The other sugars detected in all samples included maltitol, sucrose, and glucose (Table 2).

### 3.3. Fatty Acids Profile

The fatty acid profiles of the analyzed samples are presented in Table 3. The predominant saturated fatty acid was palmitic acid in all samples. Their amounts ranged from 33.36% to 39.64%. To a lesser extent, the stearic (2.86–7.32%) and lignoceric (2.33–4.956%) were also found in samples. The predominant unsaturated fatty acids in *E. planum* were linoleic acid (35.43–35.79%) and α-linolenic (7.94–8.63%). Statistical analysis revealed no significant differences in the levels of these fatty acids between the EPF and EPT parts (*p* < 0.05). Whereas, *E. billardieri* showed the highest level of oleic acid EBT (16.55%) and EBF (20.70%). Unsaturated fatty acids play an important role in body functions such as neuroprotection, antioxidant, and anti-inflammatory properties [20]. According to the findings of Sardari et al. [21], oleic and palmitic acids are the primary fatty acids present in *E. billardieri*. Additionally, oleic acid has also been detected in the fatty acid compositions of *E. maritimum* and *E. foetidum* [22,23]. Marčetić et al. [24] reported that linoleic acid (24.4%) and palmitic acid (19.9%) were the two main constituents of the volatile fraction of the chloroform extract from the aerial parts of *E. palmatum*.

Furthermore, the aerial parts of both *E. maritimum* and *E. campestre* were found to be rich in fatty acids, with *E. maritimum* containing 5.26% palmitic acid and 41.88% oleic acid and *E. campestre* comprising 5.25% palmitic acid and 41.97% oleic acid [17].

### 3.4. Elemental Analysis

The results of the elemental analysis of the studied samples are presented in Table 4. The results indicated that K, Ca, Mg, and P are the predominant macroelements. The concentrations of K (2.00%), Ca (1.72%), Mg (0.38%), and P (0.25%) in EBF were significantly higher than those in EBT, which had concentrations of K (1.38%), Ca (0.57%), Mg (0.23%), and P (0.14%). Notably, the concentrations of K and P exhibited differing trends between the flower and thorn tissues in *E. planum*.

In terms of micro-elements, the highest concentrations were detected in EBF, with the following descending order: Fe (178.1 ppm) > Mn (46.9 ppm) > Zn (18.8 ppm) > B (16.9 ppm). In EPF, the descending order was Fe (363.6 ppm) > Mn (148.9 ppm) > B (36.0 ppm) > Zn (28.5 ppm). These data show that the concentration of micro-elements in *E. planum* is higher than that in *E. billardieri*.

### 3.5. Volatile Compound Profile

The volatile compounds presented in *E. billardieri* and *E. planum* are shown in Table 5. The predominant volatile compounds found in EBF and EBT were sesquicineole (24.32% and 25.03%), spatulenol (20.69% and 14.37%), trans-chrysanthenyl acetate (11.95% and 11.84%), mesitylene (2.44% and 4.95%), β-elemene (0.62% and 1.88%), caryophyllene (0.43% and 1.58%), and (-)-carvone (1.55% and 0.76%). The results indicated that the contents of sesquicineole, mesitylene, β-elemene, and caryophyllene in EBF were higher than those of EBT.

Spathulenol has been identified as a significant component in the essential oil extracted from the aerial parts of *E. maritimum* [25], *E. campestre*, and *E. palmatum* [26]. Additionally, in the aerial parts of *E. bornmuelleri*, spathulenol and sesquicineole were found to be the predominant components [27]. Furthermore, spathulenol, alongside α-bisabolol, has been reported as a major compound in other species of *Eryngium* [28].

Trans-chrysanthenyl acetate was identified as the compound with the highest concentration in *E. planum*, particularly in EPF (34.99%), which exhibited higher contents than EPT (31.55%). Other notable compounds found in EPT and EPF included β-selinene (13.07% and 11.34%), β-elemene (7.51% and 7.24%), and (E)-β-famesene (7.00% and 1.42%), respectively. β-elemene, α-pinene, trans-β-farnesene, and cis-chrysanthenyl acetate have previously been identified as the main compounds in the essential oil of *E. planum* [10]. Andreica et al. [9] reported that the essential oil of *E. planum*, extracted using ultrasound-assisted hydrodistillation, primarily consists of β-copaene, cis-chrysanthenyl acetate, (E)-β-farnesene, γ-gurjunene, caryophyllene, germacrene B, and β-selinene. Furthermore, volatile compounds from *E. planum* extracted through HS-SPME showed a composition in which cis-chrysanthenyl acetate was the most abundant (30.39%), followed by (E)-β-farnesene, β-elemene, caryophyllene, β-selinene, δ-cadinene, β-copaene, and α-pinene [9].

### 3.6. Analysis of Phenolic Compounds

The identified phenolic compounds, along with retention times and concentrations, are summarized in Table 6. A total of 30 to 34 distinct compounds were identified in the samples. Notably, the isomer of sagerinic acid, a phenolic acid, was distinguished from other components at a retention time of 14.8 min, showing the highest concentrations of 3093.9 and 3890.2 ppm in EBT and EBF, respectively. Following this, dimeric caffeoylquinic acid, another phenolic acid, demonstrated significant concentrations of 2169.2 and 1929.9 ppm, while acetyl-diferuloyl sucrose, classified as a phenolic glycoside, showed concentrations of 858.8 and 738.4 ppm. Additionally, the compound aromadendrin-6-C-β-D-glucopyranosyl-7-O-[β-D-apiofuranosyl-(1→2)]-O-β-D-glucopyranoside was detected at concentrations of 682.1, 622.2 ppm, alongside 4-feruloyl-5-caffeoylquinic acid with concentrations of 406.9 and 365.2 ppm, both of which were categorized under flavonoids in EBT and EBF, respectively. The data also reveal variation in compound concentrations between EBF and EBT, with other identified compounds present in minor amounts, ranging from 116.0 to 497.1 ppm.

Samples of EPT and EPF exhibited a diverse range of compounds. Notably, dimeric caffeoylquinic acid (752.8 and 1093.0 ppm) and quercetin-3-O-hexoside-7-O-rhamnoside from the phenolic acids class (604.4 and 1092.7 ppm) were predominant, registering the highest concentrations in samples EPT and EPF, respectively. Other noteworthy compounds included quercetin-3-O-glucosylrutinoside (278.0 ppm), catechin dimer (248.4 ppm), kaempferol 3,7-di-o-hexoside (473.4 ppm), and kaempferol-O-rhamnodihexoside (356.6 ppm) from the flavonoids class, observed at elevated concentrations in the EPT samples. Conversely, sample EPF revealed the sagerinic acid isomer from the phenolic acids class (791.5 ppm) and kaempferol-rhamnose-hexose-rhamnose (782.2 ppm), as well as rutin (545.6 ppm), which are classified as flavonoids, were among the most abundant compounds. The findings of this study are consistent with previously published data [6,28,29]. Moreover, these results indicate that phenolic acids and flavonoids are the main phenolic compounds in both species. These plant-derived compounds possess biological activities including antioxidant, antidiabetic, anti-inflammatory, and anticancer effects [30]. Additionally, studies showed that flavonoids and phenolic acids present in white peony tea [31], *Staphylea bumalda* and *Staphylea holocarpa* plants [32], and tea leaves [33] have a key role in flavor formation.

### 3.7. Amino Acid Profile

In the analyzed samples, a total of 21 amino acids were identified (Table 7). Our findings indicate that the predominant essential amino acids in EBT and EBF were lysine (731.0 and 838.8 mg/100 gdw), arginine (155.8 and 393.5 mg/100 gdw), isoleucine (109.4 and 312.3 mg/100 gdw), and leucine (116.6 and 295.2 mg/100 gdw). Maoz et al. [34] reported that leucine, isoleucine, and valine contribute significantly to the synthesis of volatile compounds responsible for odor and aroma in plants.

Among the major non-essential amino acids, methionine sulfoxide (637.8 and 1512.1 mg/100 gdw), asparagine (710.5 and 840.1 mg/100 gdw), and glutamine (732.8 and 824.8 mg/100 gdw) were predominant in EBT and EBF, respectively. In the EPT and EPF, essential amino acids such as lysine (368.0 and 529.2 mg/100 gdw), threonine (276.0 and 217.0 mg/100 gdw), and arginine (196.2 and 241.80 mg/100 gdw) were also prevalent, alongside non-essential amino acids like glutamic acid (104.9 and 219.9 mg/100 gdw) and proline (1301.5 and 1293.0 mg/100 gdw g). Furthermore, it was observed that valine and phenylalanine were present in higher concentrations in *E. planum* than in *E. billardieri*. Previous research has shown that the aerial parts of *E. maritimum* and *E. campestre* contain significant levels of essential amino acids, including leucine, lysine, and arginine, and are rich in non-essential amino acids such as glutamic acid, glycine and proline [17].

As shown in Table 7, the amino acids levels in the thorns were lower than those in the flowers. Furthermore, the concentrations of essential and non-essential amino acids in *E. billardieri* were nearly equivalent; in contrast, *E. planum* exhibited significantly higher levels of non-essential amino acids compared to essential amino acids.

### 3.8. Antioxidant Activity and TPC of Samples

**Figure 1:**
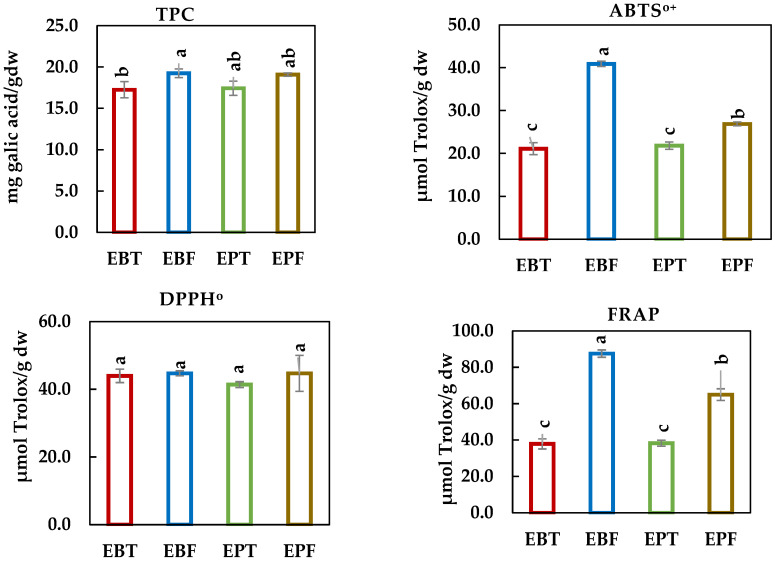
Antioxidant activities (ABTS^o+^, DPPH^o^, and FRAP) and total phenolic content (TPC) of *E. billardieri* flowers (EBF), *E. billardieri* thorns (EBT), *E. planum* flowers (EPF), and *E. planum* thorns (EPT). Data are mean ± standard deviation (n = 3). Different lowercase letter on the columns are significantly different (*p* < 0.05).

The total phenolic content and antioxidant capacities of the studied samples were evaluated using the ABTS^o+^, DPPH^o^, and FRAP assays, with the results summarized in Figure 1. The highest TPC was observed in EBF (19.25 mg GAE/gdw), while the lowest was recorded for EBT at 17.25 mg GAE/gdw. Notably, there were no significant differences in phenolic content between EPF (19.09 mg GAE/gdw) and EPT (17.44 mg GAE/gdw).

ABTS^o+^ assay—BF demonstrated the highest ABTS^o+^ scavenging activity, with a value of 40.90 µmol Trolox/gdw, followed by EPF at 26.89 µmol Trolox/gdw. Thorn extracts from both EBT and EPT exhibited comparative lower activities, recording values which showed values of 21.10 and 21.82 µmol Trolox/gdw, respectively.

DPPH^o^ assay—No significant differences were observed among the studied samples (*p* < 0.05), as all exhibited similar antioxidant activities, with values ranging from 41.39 to 44.73 µmol Trolox/gdw. Thorn extracts from both species showed slightly lower activities, with EBT at 43.97 µmol Trolox/gdw and EPT at 41.39 µmol Trolox/gdw.

FRAP assay—The results indicated a more pronounced variation in antioxidant capacity among the samples. EBF exhibited the highest ferric reducing antioxidant power, with a value of 87.53 µmol Trolox/gdw, while EPF followed closely at 64.96 µmol Trolox/gdw. The thorn extracts, EBT (37.89 µmol Trolox/gdw) and EPT (38.30 µmol Trolox/gdw), exhibited significantly lower activities compared to the flower parts (*p* < 0.05). These data indicate that both species, particularly EBF, are rich in bioactive compounds and exhibited high antioxidant activities. The results of this present study demonstrate that phenolic acids and flavonoieds are the major phenolic constituents in both species (Table 6) and that the levels of these compounds correlate strongly with antioxidant activity [35].

Previous research conducted by Daneshzadeh et al. [4] reported the TPC of *E. billardieri* extract to range from 10.71 to 33.38 mg GAE/gdw of extract, with antioxidant activity ranging between 17.25 and 51.63%. Furthermore, Mahmoudi et al. [10] investigated the effect of different drying methods on the essential oil content, total phenolic and flavonoid content, and antioxidant capacity of *E. planum*. They noted that total phenolic contents varied from 33.88 to 66.62 mg GAE/gdw of extract, and antioxidant activities were characterized by IC_50_ values from 192.66 to 844.31 µg/mL across different drying methods. Marčetić et al. [24] reported a TPC of 29.0 mg GAE/gdw for the methanolic extract of the aerial parts of *E. palmatum*, which is consistent with our findings. They also emphasized the potential of *Eryngium* species as natural source of antioxidants.

Following a comprehensive analysis of the chemical composition of the two studied species, some beneficial compounds, including essential fatty acids, amino acids, minerals, protein, phenolic compounds, organic acids, and volatile compounds were identified. In addition, our data showed that the flower part of both species possessed higher concentrations of useful compounds relative to their thorn counterparts. Therefore, to explore practical applications, we prepared herbal tea from flowers of *E. planum* and *E. billardieri*. Subsequently, color analysis and sensory evaluation were conducted.

### 3.9. Color

**Figure 2:**
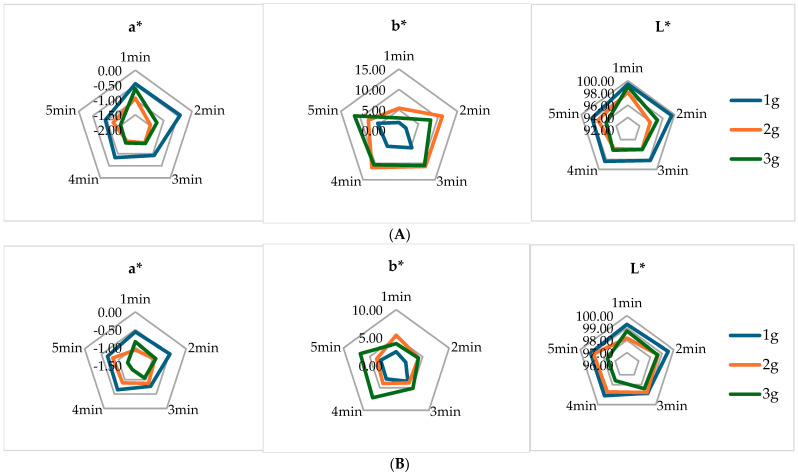
Color parameters of the produced herbal teas. (**A**) *E. planum* herbal teas; (**B**) *E. billardieri* herbal teas.

Figure 2 illustrates the color values of two herbal tea types, EPF and EBF, emphasizing the differences attributable to tea content and infusion time. Statistical analysis of the L* and a* values indicated a significant decrease with increasing tea quantity (*p* < 0.001), whereas the effect of infusion time was less pronounced (*p* < 0.05). At the maximum tea content of 3 g and an infusion duration of 5 min, the EPF tea exhibited a slightly higher lightness value (L* = 97.68) compared to EBF tea (L* = 95.69), indicating that EPF tea was lighter. Notably, EPF tea also demonstrated higher b* values, suggesting a greater intensity of blue tones; *E. planum*, known as blue eryngo, displays blue inflorescences and is commonly utilized as an ornamental plant [10]. In contrast, EBF tea exhibited the highest a* value (−1.48), indicating a slightly greener hue. Additionally, EBF tea possessed higher yellowness values, which supports the findings from sensory evaluations that indicate lower color intensity. Both tea infusions presented green-yellow hues, which may be influenced by plant pigments, including chlorophyll, carotenoids, and anthocyanins. Suzuki et al. [36] reported that chlorophyll plays a crucial role in determining the quality of green tea.

### 3.10. Sensory Evaluation

The sensory analysis at the visual, olfactory, and gustatory phases of the final herbal tea was performed. The mean scores revealed that samples with higher quantities of tea and longer infusion times received the highest scores during the visual phase (color intensity) in both herbal teas (*p* < 0.05). Importantly, none of the samples exhibited turbidity. Among the EBF tea samples, those containing 3 g of tea infused for 5 min achieved the highest scores for vegetable and herbal aroma during the gustatory phase (*p* < 0.05). Whereas, in the olfactory phase, significant differences (*p* < 0.05) were observed in the scores for floral flavor in EPF tea, with 2 g tea and infused time 2 min. However, no significant differences (*p* < 0.05) were noted in the scores for other sensory properties. It should be mentioned that panelists identified a balsamic aroma in the EBF tea and a honey-like scent in the EPF tea during the olfactory phase. Furthermore, in the gustatory phase, the EBF teas were characterized by balsamic, honey, irritating, and astringent qualities, while the EPF teas exhibited a slight bitterness.

Previous studies have shown that specific aroma compounds in herbal teas contribute to distinct types of aroma. For instance, β-elemene, β-cadinene, and β-selinene are typically associated with herbal scents [37], while phenylacetaldehyde is known for imparting a honey odor [38]. Moreover, α-cubebene is recognized for its woody, fruity, and floral notes [39] and α-farnesene, a key stress-related compound, contributes a floral and woody aroma [40]. These aromatic compounds are present in *E. billardieri* and *E. planum*, as detailed in Table 5. Astringency is typically attributed to the presence of tannins or polyphenols, and it can be considered a desirable characteristic in tea [41].

## 4. Conclusions

The results of the present study demonstrate that both investigated *Eryngium* species possess valuable nutritional compositions, characterized by elevated total phenolic content (TPC) and significant antioxidant activities. Notable variations in the chemical compositions were observed between the two species, with the flower parts showing a higher concentration of bioactive compounds compared to the thorn parts. *E. billardieri* was identified as a rich source of essential amino acids, while *E. planum* demonstrated higher levels of protein, sugar, and mineral content. Moreover, *E. planum* contained elevated concentrations of linoleic acid (omega-6) and α-linolenic acid (omega-3), whereas oleic acid (omega-9) was found in greater abundance in *E. billardieri*. Key compounds identified in *E. billardieri* included the isomer of sagerinic acid and dimeric caffeoylquinic acid, whereas *E. planum* was characterized by dimeric caffeoylquinic acid and quercetin-3-O-hexoside-7-O-rhamnoside. Notably, dimeric caffeoylquinic acid was a major component in both species, with a higher concentration recorded in *E. billardieri*. Given their favorable characteristics, including positive perception scores related to color, aroma, and overall taste, it is recommended to utilize *Eryngium* flower parts as functional ingredients in herbal teas. The presence of volatile and phenolic compounds in both species further supports their potential as functional ingredients and flavor enhancers. Additionally, the essential fatty acids, amino acids, and various minerals present in these species enhance their suitability for improving the nutritional profile of food products. The high total phenolic content and strong antioxidant properties underscore their potential applications in the pharmaceutical industry.

## Figures and Tables

**Table 1:** Analysis of the proximate compositions of the aerial parts of two studied plants *.

Parameters (%)	*E. billardieri*		*E. planum*	
	EBT	EBF	EPT	EPF
Dry matter	93.2 ± 1.0 a	93.3 ± 0.0 a	92.0 ± 0.1 a	93.1 ± 0.2 a
Moisture	6.8 ± 1.0 a	6.7 ± 0.0 a	8.1 ± 0.1 a	7.0 ± 0.2 a
Ash	4.9 ± 0.4 c	9.0 ± 0.0 b	10.3 ± 0.17 a	10.0 ± 0.1 a
Protein	4.5 ± 0.2 d	8.3 ± 0.0 c	10.4 ± 0.1 b	11.2 ± 0.0 a
Fat	0.5 ± 0.1 b	1.8 ± 0.2 a	2.0 ± 0.0 a	1.8 ± 0.1 a
Carbohydrates	83.3 ± 0.4 a	71.9 ± 0.1 b	71.5 ± 0.1 b	70.1 ± 0.2 c
Crude fiber (%)	50.9 ± 3.6 a	34.5 ± 1.6 b	25.0 ± 2.0 c	22.0 ± 3.1 d
Neutral detergent fiber (%)	71.8 ± 0.9 a	51.5 ± 2.4 b	44.5 ± 0.0 c	45.0 ± 0.8 c
Acid detergent fiber (%)	48.8 ± 0.5 a	35.7 ± 0.3 b	29.7 ± 0.0 c	29.7 ± 0.9 c

* Values are mean ± standard deviation (n = 3). Different lowercase letters in the same row are significantly different (*p* ˂ 0.05); EBF: *E. billardieri* flowers; EBT: *E. billardieri* thorns; EPF: *E. planum* flowers; EPT: *E. planum* thorns.

**Table 2:** Organic acids and sugars profiles of two studied plants (mg/gdw) *.

Organic Acid	*E. billardieri*		*E. planum*	
	EBT	EBF	EPT	EPF
Citric acid	4.7 ± 0.4 b	7.2 ± 0.1 a	8.6 ± 1.0 a	8.3 ± 0.1 a
Tartaric acid	0.7 ± 0.1 c	0.6 ± 0.1 c	2.7 ± 0.4 a	1.9 ± 0.2 b
Malic acid	12.2 ± 0.9 c	13.8 ± 0.6 b	15.6 ± 0.3 a	12.8 ± 0.1 bc
Quinic acid	12.0 ± 2.6 c	16.3 ± 0.5 a	16.0 ± 0.3 a	13.3 ± 0.0 ab
Sugars				
Sucrose	23.8 ± 1.9 c	22.8 ± 0.54 c	49.6 ± 1.2 a	33.8 ± 0.7 b
Maltitol	62.1 ± 0.9 c	62.4 ± 1.7 c	70.7 ± 1.0 a	66.3 ± 0.8 b
Glucose	14.8 ± 1.1 c	19.2 ± 0.5 b	24.1 ± 0.6 a	23.5 ± 0.5 a
Fructose	44.7 ± 2.8 d	56.4 ± 0.9 c	79.6 ± 1.2 a	72.5 ± 0.4 b
Total	145.6	160.9	224.2	196.3

* Values are mean ± standard deviation (n = 3). Different letters in the same rows indicate significant differences (*p* < 0.05). EBF: *E. billardieri* flowers; EBT: *E. billardieri* thorns; EPF: *E. planum* flowers; EPT: *E. planum* thorns.

**Table 3:** Fatty acids profile of the studied plants (%) *.

Fatty Acid	*E. billardieri*		*E. planum*	
SFA	EBT	EBF	EPT	EPF
C6:0 (Caproic)	0.52 ± 0.06 a	0.21 ± 0.03 b	0.62 ± 0.06 a	0.55 ± 0.12 a
C8:0 (Caprylic)	0.77 ± 0.07 a	0.4 ± 0.15 b	1.09 ± 0.12 a	1.00 ± 0.17 a
C12:0 (Lauric)	0.89 ± 0.42 a	0.49 ± 0.13 a	0.23 ± 0.15 a	tr
C14:0 (Myristic)	1.35 ± 0.26 a	1.09 ± 0.17 ab	0.44 ± 0.06 c	0.82 ± 0.11 bc
C15:0 (Pentadecanoic)	1.41 ± 0.27 a	0.93 ± 0.04 b	0.80 ± 0.09 b	0.57 ± 0.15 b
C16:0 (Palmitic)	39.64 ± 2.62 a	36.19 ± 1.03 ab	33.36 ± 1.47 b	34.01 ± 0.20 b
C17:0 (Heptadecanoic)	1.06 ± 0.19 a	0.62 ± 0.14 b	0.76 ± 0.15 ab	0.50 ± 0.03 b
C18:0 (Stearic)	7.32 ± 0.90 a	4.96 ± 0.79 b	2.86 ± 0.39 c	3.09 ± 0.0.25 c
C20:0 (Arachidic)	tr	0.84 ± 0.11 ab	0.62 ± 0.12 b	0.98 ± 0.12 a
C21:0 (Heneicosanoic)	tr	tr	tr	0.17 ± 0.05
C22:0 (Behenic)	1.20 ± 0.03 a	1.43 ± 0.36 a	1.61 ± 0.04 a	1.57 ± 0.06 a
C23:0 (Tricosanoic)	tr	0.55 ± 0.49 a	1.25 ± 0.24 a	0.99 ± 0.25 a
C24:0 (Lignoceric)	2.57 ± 0.88 ab	2.97 ± 0.51 ab	4.56 ± 0.60 a	2.33 ± 1.10 b
MUFA				
C18:1 c9/C18:1 n9 (Oleic)	16.55 ± 3.02 a	20.70 ± 0.68 a	7.47 ± 0.86 b	9.32 ± 0.40 b
n-6 PUFA				
C18:2 n6 c (Linoleic)	17.09 ± 1.45 c	22.22 ± 0.08 b	35.43 ± 0.72 a	35.79 ± 0.79 a
n-3 PUFA				
C18:3 n3 (α-Linolenic)	5.98 ± 0.93 b	5.70 ± 0.36 b	8.63 ± 0.17 a	7.94 ± 0.15 a

* Values are mean ± standard deviation (n = 3). Different lowercase letters in the same row are significantly different (*p* ˂ 0.05); EBF: *E. billardieri* flowers; EBT: *E. billardieri* thorns; EPF: *E. planum* flowers; EPT: *E. planum* thorns; SFA: Saturated fatty acids; MUFA: Monounsaturated fatty acids; n-3 PUFA: Omega-3 polyunsaturated fatty acids; n-6 PUFA: Omega-6 polyunsaturated fatty acids. tr: trace amounts detected.

**Table 4:** Elemental analysis of the two studied plants *.

Mineral	*E. billardieri*		*E. planum*	
	EBT	EBF	EPT	EPF
Macro (%)				
Ca	0.579 ± 0.017 b	1.727 ± 0.071 a	0.835 ± 0.040 b	1.983 ± 0.190 a
K	1.386 ± 0.073 d	2.009 ± 0.037 c	3.229 ± 0.010 a	2.918 ± 0.020 b
Mg	0.238 ± 0.012 d	0.389 ± 0.002 a	0.318 ± 0.010 b	0.278 ± 0.020 c
Na	0.003 ± 0.001 b	0.005 ± 0.001 b	0.012 ± 0.010 a	0.010 ± 0.001 a
P	0.135 ± 0.014 d	0.249 ± 0.005 c	0.459 ± 0.001 a	0.382 ± 0.001 b
Micro (ppm)				
As	0.14 ± 0.01 c	0.23 ± 0.01 b	0.17 ± 0.20 c	0.30 ± 0.01 a
B	10.95 ± 1.58 c	16.94 ± 0.20 b	37.25 ± 1.00 a	36.05 ± 1.11 a
Cd	0.01 ± 0.01 a	0.03 ± 0.01 a	0.061 ± 0.001 a	0.03 ± 0.01 a
Cu	3.32 ± 0.25 c	4.54 ± 0.18 b	9.37 ± 0.44 a	9.99 ± 0.47 a
Fe	32.35 ± 2.03 d	178.15 ± 3.64 b	95.52 ± 3.21 c	363.57 ± 6.16 a
Mn	34.49 ± 2.06 c	46.97 ± 0.68 b	142.47 ± 5.01 a	148.89 ± 3.23 a
Pb	0.32 ± 0.04 c	0.56 ± 0.01 b	0.55 ± 0.14 b	0.97 ± 0.02 a
Se	0.41 ± 0.02 a	0.63 ± 0.18 a	0.69 ± 0.20 a	0.51 ± 0.05 a
Zn	10.27 ± 1.06 c	18.87 ± 0.26 b	26.75 ± 0.73 a	28.51 ± 2.49 a

* Values are mean ± standard deviation (n = 3). Different lowercase letters in the same row are significantly different (*p* ˂ 0.05); EBF: *E. billardieri* flowers; EBT: *E. billardieri* thorns; EPF: *E. planum* flowers; EPT: *E. planum* thorns.

**Table 5:** Volatile compound analysis of two studied plants *.

Compound (%)	R_t_ (min)	KI (Exp.)	KI (Lit)	EBT	EBF	EPT	EPF
α-Pinene	8.76	933	939	n.d.	n.d.	0.20	n.d.
Heptanal	7.77	900	903	0.25	0.16	0.2	0.46
Mesitylene	10.57	992	996	2.44	4.95	0.11	n.d.
Octanal	10.86	1002	1001	0.92	0.56	1.14	1.38
Limonene	11.71	1029	1031	0.13	0.20	0.17	n.d.
Benzeneacetaldehyde	12.11	1042	1044	n.d.	n.d.	n.d.	0.14
(E)-2-Octenal	12.58	1057	1056	0.25	0.255	n.d.	0.25
(E)-2-Octen-1-ol	12.85	1065	1064	n.d.	n.d.	n.d.	0.17
1-Octanol	12.97	1069	1070	0.53	0.36	0.65	1.88
2-Nonanone	13.57	1089	1091	0.41	0.26	0.88	0.92
γ-Terpinene	13.76	1095	1089	0.4	0.84	n.d.	n.d.
1,5,7-Octatrien-3-ol, 3,7-dimethyl-	13.97	1101	1106	n.d.	n.d.	0.29	0.3
Nonanal	14.01	1103	1102	0.37	0.34	0.20	n.d.
Octanoic acid, methyl ester	14.58	1122	1120	n.d.	n.d.	0.12	n.d.
(E)-2-Nonenal	15.69	1257	1239	0.19	n.d.	0.12	0.25
(E)-2-Cyclohexen-1-ol, 1-methyl-4-(1-methylethenyl)	15.80	1162	1132	n.d.	n.d.	0.55	1.11
Octanoic acid	16.00	1169	1169	0.66	0.19	0.80	1.67
(-)-Carvone	18.19	1243	1243	1.55	0.76	0.38	0.62
Linalyl acetate	18.33	1248	1248	n.d.	0.14	0.09	n.d.
trans-Chrysanthenyl acetate	18.58	1257	1239	11.95	11.84	31.55	34.99
(E)-2-Decenal	18.69	1257	1239	0.56	0.55	0.22	0.61
(E)-Cinnamaldehyde	19.0	1266	1268	0.26	n.d.	n.d.	n.d.
Bornyl acetate	19.37	1284	1285	n.d.	0.17	n.d.	n.d.
trans-Pinocarvyl acetate	19.66	1295	1297	0.69	1.41	n.d.	n.d.
Copaene	21.96	1378	1377	n.d.	0.22	0.38	n.d.
β-Cubebene	21.96	1378	1372	n.d.	0.24	0.24	n.d.
β-Elemene	22.30	1391	1392	0.615	1.88	7.51	7.24
Caryophyllene	23.13	1423	1418	0.42	1.58	0.21	n.d.
β-gurjunene	23.44	1435	1432	n.d.	0.23	n.d.	n.d.
(E)-β-Famesene	23.87	1452	1458	n.d.	0.26	7	1.42
Humulene	24.05	1459	1455	n.d.	0.43	0.19	n.d.
α-Curcumene	24.64	1481	1486	n.d.	0.53	n.d.	n.d.
β-Selinene	24.92	1492	1489	0.5	1.48	13.07	11.34
β-Bisabolene	25.31	1508	1500	0.39	1.18	n.d.	n.d.
Sesquicineole	25.45	1514	1516	24.33	25.03	1.29	1.00
β-Cadinene	25.59	1520	1520	0.54	0.29	n.d.	n.d.
(-)-β-Panasinsen	25.68	1523	1521	n.d.	n.d.	0.33	0.29
β-Sesquiphellanderene	25.71	1531	1521	0.39	0.69	n.d.	n.d.
Sesquicineole	25.85	1525	1524	0.45	0.26	n.d.	n.d.
Elemol	26.311	1529	1520	n.d	n.d	0.7	1.25
Spatulenol	27.055	1570	1572	20.69	14.37	0.57	0.97
Carotol	27.68	1580	1578	0.38	0.455	n.d.	n.d.
2-Furanmethanol, tetrahydro-α, α,5-trimethyl-5-(4-methyl-3-cyclohexen-1-yl)	28.875	1606	1598	1.33	0.73	n.d.	n.d.
α-Bisabolol	29.533	1658	1656	0.485	0.54	n.d.	n.d.

* Values are mean ± standard deviation (n = 3). EBF: *E. billardieri* flowers; EBT: *E. billardieri* thorns; EPF: *E. planum* flowers; EPT: *E. planum* thorns; R_t_ = Retention time; KI = Kovats index; Exp = Experimental; Lit = Literature; n.d.: not detected.

**Table 6:** Phenolic compounds analysis of the two studied plants *.

No.	Compounds (ppm)	[M-H]− (*m*/*z*)	MS^2^	R_t_ (min)	UV-Vis (nm)	EBT	EBF	EPT	EPF
1	Sagerinic acid isomer	719	359; 197; 135	3.1	320	352.3	273.8	294.5	266.4
2	Aromadendrin-6-C-β-D-glucopyranosyl-7-O-[β-D-apiofuranosyl-(1→2)]-O-β-Dglucopyranoside	743	371; 209	4.2	320	453.6	415.0	317.7	325.1
3	3-Feruloyl-5-caffeoylquinic acid	367	367; 190	4.8	320	235.4	233.0	197.8	189.7
4	Aromadendrin-6-C-β-D-glucopyranosyl-7-O-[β-D-apiofuranosyl-(1→2)]-O-β-Dglucopyranoside	743	371; 209	5.3	320	682.1	622.2	405.6	437.9
5	Sinapic acid-O-glucoside	385	367; 223; 129	5.7	320	147.8	143.5	270.6	350.7
6	4-Feruloyl-5-caffeoylquinic acid	367	367; 190	6.1	320	406.9	365.2	228.6	317.1
7	Dimeric caffeoylquinic acid	707	353; 190	6.6	320	2169.2	1929.9	752.8	1093.0
8	unknown metabolite	771	385	7.8	320	497.1	425.8	481.2	818.8
9	Galloyl paeoniflorin	399	380; 205; 129	8.1	320	208.3	183.8	n.d.	189.5
10	Apigenin-6,8-di-C-glycoside (Vicenin-2)	593	575; 503; 473; 383; 353; 297	8.7	320	231.3	125.3	n.d.	n.d.
11	Quercetin-3-O-glucosylrutinoside	771	625; 447; 301	8.8	360	n.d.	n.d.	278.0	111.1
12	Acetyl-diferuloyl sucrose	735	367; 190	9.2	320	858.8	738.4	218.4	540.0
13	Kaempferol-O-rhamnodihexoside	755	575; 489; 393; 327; 285	9.3	360	n.d.	122.9	356.6	325.6
14	Kaempferol 3-O-(2″-O-hexosyl)hexoside-7-O-rhamnoside	755	609, 285	9.7	360	n.d.	n.d.	226.2	164.2
15	Kaempferol-rutinoside/Kaempferol 3-coumaroylglucoside	593	447; 285	10.5	360	375.7	339.8	122.7	90.6
16	Quercetin-3-O-hexoside-7-O-rhamnoside	609	447; 301	10.8	360	241.3	116.0	604.4	1092.7
17	Luteolin hexoside hexoside/Quercetin 3-O-(6″-O-rhamnosyl)glucoside (Rutin)	609	343; 301	10.9	360	308.0	186.4	190.4	545.6
17.1	Kaempferol 3,7-di-O-hexoside	609	285	11	360	n.d.	n.d.	473.4	n.d.
18	Catechin dimer	579	289; 245; 203	11.3	360	n.d.	n.d.	248.4	178.4
19	Kaempferol derivative	635	489; 431; 285	11.4	360	417.5	397.9	n.d.	n.d.
20	Kaempferol-rhamnose-hexose-rhamnose	739	593; 431; 285	11.8	360	n.d.	n.d.	378.3	782.2
21	Rosmarinic acid-4-O-glucoside	521	359; 197	12.4	320	275.8	n.d.	327.9	395.6
22	Kaempferol derivative	781	635; 593; 473; 431; 285	12.7	360	n.d.	n.d.	n.d.	424.0
23	Kaempferol-rutinoside/Luteolin-7-O-rutinoside	593	285	12.9	360	n.d.	286.0	n.d.	n.d.
24	Ellagic acid-O-deoxyhexoside	447	301	13	360	342.1	329.0	424.5	555.2
25	Kaempferol derivative	781	635; 593; 431; 285	13.6	360	n.d.	n.d.	n.d.	237.8
26	1,4-di-O-caffeoylquinic acid/3,5-Di-O-caffeoylquinic acid	515	353; 299; 255; 173	14.1	320	246.1	171.8	n.d.	n.d.
27	Kaempferol-3-O-acetylhexoside	489	285	14.4	360	180.6	165.6	242.8	232.7
28	Sagerinic acid isomer	719	359	14.8	320	3093.9	3890.2	185.2	791.5
29	Rosmarinic acid glucuronide	535	359; 197	14.9	320	221.8	165.3	135.6	217.1
30	Kaempferol-3-O-rhamnoside	431	285	15	360	n.d.	n.d.	n.d.	206.1
31	Methylellagic acid acetyl hexose	519	315; 357	15.1	360	201.4	379.9	n.d.	n.d.
32	unknown metabolite	747	373	17.9	360	610.6	163.2	n.d.	n.d.
33	Kaempferol-5-O-rhamnoside	431	285	18.6	360	n.d.	n.d.	n.d.	n.d.
34	Kaempferol-rhamnose-hexose-rhamnose	739	593; 435; 285	23.3	360	256.6	126.7	n.d.	463.3

* EBF: *E. billardieri* flowers; EBT: *E. billardieri* thorns; EPF: *E. planum* flowers; EPT: *E. planum* thorns; R_t_ = Retention time; n.d.: not detected.

**Table 7:** Amino acids profiles of the two studied plants (mg/100 gdw) *.

Name	*E. billardieri*		*E. planum*	
	EBT	EBF	EPT	EPF
Nonessential Amino Acids				
Asparagine	710.5 ± 17.7 b	840.1 ± 3.5 a	358.5 ± 54.2 d	451.7 ± 41.6 c
Aspartic acid	94.6 ± 8.9 a	86.0 ± 7.7 ab	23.8 ± 2.0 c	49.8 ± 2.8 bc
Serine	48.0 ± 4.4 b	69.4 ± 5.3 a	28.0 ± 2.1 c	56.0 ± 2.8 b
Alanine	5.3 ± 0.7 a	51.3 ± 8.0 a	53.4 ± 18.4 a	40.4 ± 6.1 a
Glycine	tr	tr	2.1 ± 1.4 a	1.9 ± 0.6 a
Glutamine	732.8 ± 18.4 a	824.8 ± 31.3 a	318.3 ± 64.3 c	523.6 ± 4.6 b
Cysteine	4.1 ± 0.7 b	6.3 ± 0.1 a	2.9 ± 0.8 b	3.7 ± 0.1 b
Methionine sulfoxide	637.8 ± 42.0 d	1521.1 ± 4.0 a	1155.4 ± 43.7 ab	1123.2 ± 9.3 c
Glutamic acid	156.7 ± 3.2 c	190.1 ± 2.2 b	104.9 ± 4.2 d	219.9 ± 5.3 a
Proline	486.5 ± 16.0 c	741.9 ± 64.7 b	1301.5 ± 76.4 a	1293.0 ± 26.1 a
Tyrosine	99.5 ± 1.7 b	167.9 ± 6.7 a	79.4 ± 5.8 c	86.5 ± 5.3 ab
Total nonessential amino acids	2489.3	3757.0	3428.2	3849.7
Essential amino acids				
Threonine	271.7 ± 11.7 a	336.6 ± 185.3 a	196.2 ± 6.4 a	241.8 ± 1.9 a
Lysine	731.1 ± 19.0 b	838.8 ± 13.6 a	368.0 ± 20.8 d	529.2 ± 4.1 c
Histidine	25.2 ± 0.2 c	87.4 ± 3.6 a	8.1 ± 0.8 d	53.0 ± 0.9 b
Arginine	155.8 ± 12.1 d	393.5 ± 23.0 a	276.0 ± 5.6 b	217.0 ± 11.3 c
Valine	8.7 ± 1.7 c	66.3 ± 43.8 bc	133.3 ± 21.2 ab	159.3 ± 23.5 a
Methionine	tr	tr	1.68 ± 0.8	tr
Leucine	116.6 ± 9.6 b	295.2 ± 2.0 a	84.9 ± 5.8 c	85.5 ± 4.7 c
Phenylalanine	54.5 ± 4.2 b	106.3 ± 10.4 a	111.8 ± 2.9 a	123.5 ± 8.5 a
Tryptophan	147.3 ± 9.2 c	197.0 ± 1.5 a	95.5 ± 1.7 d	164.4 ± 5.8 b
Isoleucine	109.4 ± 0.1 b	312.3 ± 8.9 a	58.7 ± 14.4 c	56.5 ± 10.8 c
Total essential amino acids	2106.8	3375.3	1334.2	1630.2
Total amino acids	4596.1	7132.3	4762.4	5479.9

* Values are mean ± standard deviation (n = 3). Different lowercase letters in the same row are significantly different (*p* ˂ 0.05); EBF: *E. billardieri* flowers; EBT: *E. billardieri* thorns; EPF: *E. planum* flowers; EPT: *E. planum* thorns. tr = trace amounts detected.

## Data Availability

The data presented in this study are available on request from the corresponding author. The data are not publicly available due to privacy restrictions.

## Supplementary Materials

The following supporting information can be downloaded at https://www.mdpi.com/article/10.3390/foods14010118/s1: Table S1: Sensory evaluation form of produced herbal teas.
