# Supplementary material for: Characterization of the Different Chemical Components and Nutritional Properties of Two Eryngium Species
Source: Foods. 2025 Jan 3;14(1):118. doi: 10.3390/foods14010118 (PMC11719572; doi:10.3390/foods14010118)
Supplement: Supplementary file 1 [file foods-14-00118-s001.zip › foods-3400442-supplementary.pdf]

Table S1. Sensory evaluation form of produced herbal teas.

EBF Tea	Sensory attributes	1	2	3	4	5	6
Visual phase	Color						
	Opacity						
	Turbidity						
Olfactory phase	Vegetal						
	Herbal						
	Floral						
	Chamomile						
	Balsamic						
	Earthy						
	Other (Anise)						
Gustatory phase	Sweet						
	Vegetal						
	Herbal						
	Floral						
	Honey						
	Chamomile						
	Balsamic						
	Earthy						
	Wood						
	Irritating						
	Astringency						
	Other (Anise)						
	Other (like straw)						
	Aftertaste						

EPF Tea	Sensory attributes	1	2	3	4	5	6
Visual phase	Color						
	Opacity						
	Turbidity						
Olfactory phase	Vegetal						
	Herbal						
	Floral						
	Honey						
	Chamomile						
	Earthy						
	Other (Anise)						
Gustatory phase	Sweet						
	Bitter						
	Vegetal						
	Herbal						
	Floral						
	Chamomile						
	Earthy						
	Wood						
	Other (Anise)						
	Other (like straw)						
	Aftertaste						
